# Relationships of change in Clinical Dementia Rating (CDR) on patient outcomes and probability of progression: observational analysis

**DOI:** 10.1186/s13195-024-01399-7

**Published:** 2024-02-15

**Authors:** Pierre N. Tariot, Mercè Boada, Krista L. Lanctôt, Julie Hahn-Pedersen, Firas Dabbous, Sariya Udayachalerm, Lars Lau Raket, Yuliya Halchenko, Wojciech Michalak, Wendy Weidner, Jeffrey Cummings

**Affiliations:** 1 Banner Alzheimer’s Institute, Phoenix, AZ 85006 USA; 2 Ace Alzheimer Center Barcelona – Universitat Internacional de Catalunya, 08028 Barcelona, Spain; 3 Networking Research Center On Neurodegenerative Diseases (CIBERNED), Instituto de Salud Carlos III, 28029 Madrid, Spain; 4 Sunnybrook Research Institute, Toronto, ON M4N 3M5 Canada; 5 Novo Nordisk A/S, 2860 Søborg, Denmark; 6 Evidera, Bethesda, MD 20814 USA; 7 Clinical Memory Research Unit, Department of Clinical Sciences, Lund University, Lund, Sweden; 8 Alzheimer’s Disease International, London, SE1 4PU UK; 9 Chambers-Grundy Center for Transformative Neuroscience, UNLV, Las Vegas, NV 89154 USA

**Keywords:** Alzheimer’s disease, Progression, Activities of daily living, Neuropsychiatric features, Cognitive impairment, Institutionalization, Long-term care facility, Clinical Dementia Rating, Transition probabilities, Burden

## Abstract

**Background:**

Understanding the relationship among changes in Clinical Dementia Rating (CDR), patient outcomes, and probability of progression is crucial for evaluating the long-term benefits of disease-modifying treatments. We examined associations among changes in Alzheimer’s disease (AD) stages and outcomes that are important to patients and their care partners including activities of daily living (ADLs), geriatric depression, neuropsychiatric features, cognitive impairment, and the probabilities of being transitioned to a long-term care facility (i.e., institutionalization). We also estimated the total time spent at each stage and annual transition probabilities in AD.

**Methods:**

The study included participants with unimpaired cognition, mild cognitive impairment (MCI) due to AD, and mild, moderate, and severe AD dementia in the National Alzheimer’s Coordinating Center (NACC) Uniform Data Set (UDS) database. The associations among change in AD stages and change in relevant outcomes were estimated using linear mixed models with random intercepts. The probability of transitioning to long-term care facilities was modeled using generalized estimating equations. The total length of time spent at AD stages and annual transition probabilities were estimated with multistate Markov models.

**Results:**

The estimated average time spent in each stage was 3.2 years in MCI due to AD and 2.2, 2.0, and 2.8 years for mild, moderate, and severe AD dementia, respectively. The annual probabilities of progressing from MCI to mild, moderate, and severe AD dementia were 20, 4, and 0.7%, respectively. The incremental change to the next stage of participants with unimpaired cognition, MCI, and mild, moderate, and severe AD dementia (to death) was 3.2, 20, 26.6, 31, and 25.3%, respectively. Changes in ADLs, neuropsychiatric features, and cognitive measures were greatest among participants who transitioned from MCI and mild AD dementia to more advanced stages. Participants with MCI and mild and moderate AD dementia had increasing odds of being transitioned to long-term care facilities over time during the follow-up period.

**Conclusions:**

The findings demonstrated that participants with early stages AD (MCI or mild dementia) were associated with the largest changes in clinical scale scores. Early detection, diagnosis, and intervention by disease-modifying therapies are required for delaying AD progression. Additionally, estimates of transition probabilities can inform future studies and health economic modeling.

## Background

Alzheimer’s disease (AD) is a progressive neurodegenerative disease impacting approximately 55 million people ≥ 65 years old worldwide in 2019 [1]. AD is characterized by the presence of abnormal beta-amyloid protein accumulation in the brain and associated hyperphosphorylation of tau proteins [2]. The progression of AD from brain changes that are unnoticeable to the individual manifesting cognitive symptoms and eventually to physical disability is a continuum, encompassing unimpaired cognition, mild cognitive impairment (MCI) due to AD, and mild, moderate, and severe AD dementia [3]. The burden of AD is substantial as it significantly decreases life expectancy and leads to physical disability, may need management in a long-term care facility (i.e., institutionalization), and causes worsened quality of life, adding to health, social, and economic burden for individuals, family, and the healthcare system [4, 5].

The Clinical Dementia Rating Scale (CDR) is a clinical instrument that is widely used primarily in research/clinical trial settings to assess and standardize the staging of AD [6–9]. According to the CDR Global scores, AD stages are often defined as unimpaired cognition (CDR Global = 0), MCI due to AD (CDR Global = 0.5), mild AD dementia (CDR Global = 1), moderate AD dementia (CDR Global = 2), and severe AD dementia (CDR Global = 3). Previous studies have linked the CDR staging of AD to neuropathological changes. Higher CDR scores and use of psychotropic medications were associated with reduced activities of daily living (ADLs) and physical functioning, more severe neuropsychiatric features, and significant cognitive decline [10–15]. Difficulties in ADLs, depression, and other neuropsychiatric features occur as early as MCI due to AD and mild AD dementia. Numerous studies have indicated that more advanced CDR staging of AD, difficulties in ADLs, and neuropsychiatric features are important contributors in decisions to move patients to residential facilities [16–18].

Despite the known associations of CDR staging of AD with progressive cognitive, behavioral, and functional impairment, few prior studies have explored the CDR-based stage-to-stage transition probabilities and the impact of change in AD stages on these outcomes [19]. Such estimates provide an important context for time-to-worsening endpoints based on CDR Global scores that have recently been reported from trials of high-clearance anti-amyloid immunotherapies [20, 21]. A commonly used estimate of stage-to-stage transition probabilities is based on the Neumann et al. study, which calculated the transition probabilities using the Consortium to Establish a Registry for Alzheimer’s Disease (CERAD) data [22, 23]. However, those transition probabilities were based on data collected from 1986 to 1995, which may not represent the AD population receiving contemporary care of AD [19, 22, 24]. Furthermore, there are limited data from longitudinal studies examining the associations among validated measures of AD disease progression and transition to long-term care facilities.

In this study, we estimated the average time spent at AD disease stages and annual transition probabilities by AD disease stages, including MCI due to AD and mild, moderate, and severe AD dementia. Additionally, we evaluated the associations among changes in AD stages and changes in relevant outcomes that are important to clinicians, patients, and their care partners, including ADLs measured by the National Alzheimer’s Coordinating Center Functional Assessment Scale (NACC-FAS), geriatric depression quantified by the Geriatric Depression Scale (GDS), the Neuropsychiatric Inventory Questionnaire (NPI-Q), the Mini-Mental State Exam (MMSE), and the Montreal Cognitive Assessment (MoCA). Lastly, we explored how the CDR stage of AD at the initial visit contributed to the likelihood of transition to long-term care facilities and to death over time.

## Methods

### Data source

This study used publicly available data from the NACC Uniform Data Set (UDS). The NACC was established in 1999 and captures data of participants from 40 Alzheimer’s Disease Research Centers (ADRCs) across the USA that are supported by the National Institute on Aging (NIA) [25, 26]. One of the variables that NACC tracks is institutionalization, which refers to the residence in long-term care facilities such as nursing homes or assisted living facilities. Participants enrolled in the NACC UDS reflect clinical referrals at ADRCs, self-referral, referral by family members, or active recruitment through community organizations. Information on demographics, clinical evaluations, neuropathological data, and magnetic resonance imaging was collected during clinic visits, home visits and telephone calls, and autopsy findings. Depending on a given ADRC protocol, the AD diagnosis was made by either a consensus team or a single physician (the one who conducted the examination). The specific outcomes presented here were collected at the participant visits.

### Study design

Adult participants were included in the study if they visited any of the affiliated ADRCs, were cognitively unimpaired, clinically diagnosed with MCI due to AD or dementia due to AD of any severity, and had at least one annual visit in the database. Participants with MCI or dementia due to causes other than AD were excluded. We excluded participants whose CDR staging of AD significantly differed from their clinical diagnosis and those with MCI or dementia due to AD who reverted to lower AD stages in subsequent visits (i.e., had a decreased CDR score over time, which is probably attributed to data entry errors). The final analytical sample included 28,220 participants with longitudinal data from up to six visits. Participants were classified according to CDR Global scores (CDR Global) into the following categories: unimpaired cognition (CDR Global = 0), MCI due to AD (CDR Global = 0.5), mild (CDR Global = 1), moderate (CDR Global = 2), and severe AD dementia (CDR Global = 3) [27].

### Outcomes

The outcomes included measures of ADLs, geriatric depression, neuropsychiatric features, and transition to long-term care facilities and death, all of which are deemed relevant to the patients, care partners, clinicians, and payers [28]. ADLs were measured using the 10-item NACC-FAS [29]. Each item is scored from 0 to 3 with a total score ranging from 0 to 30. Depression in older adults was quantified by GDS. The scale consists of 15 items with a total score ranging from 0 to 15. Neuropsychiatric features were assessed by NPI-Q, which includes 12 items. Each item is scored from 0 to 3 with a total score ranging from 0 to 36. Higher scores of NACC-FAS, GDS, and NPI-Q indicate greater disability. Cognition was measured using MMSE and MoCA. The MMSE was used to measure cognitive performance with 11 question domains of orientation, registration, attention and calculation, recall, and language and a total score ranging from 0 to 30. The MoCA consists of 11 domains (e.g., executive function, visuoconstructional skills) with a total score ranging from 0 to 30. Lower MMSE and MoCA scores indicate more cognitive impairment. Long-term care facility was defined as any of the following admissions: (1) assisted living, adult family home (a type of long-term care facility that provides housing, meals, and personal care services to a small group of adults, typically between 2 and 6 residents), or boarding home and (2) skilled nursing facility, nursing home, hospital, or hospice.

### Statistical analyses

Descriptive statistics (mean, standard deviation [SD], proportion) were calculated for demographics, clinical characteristics, and outcomes at baseline in the total population and stratified by CDR Global staging.

Total length of time spent at AD disease stages and the annual transition probabilities among AD stages were modeled using multistate Markov models with and without misclassification assumption. The associations of transition in AD with ADLs by NACC-FAS, GDS, and NPI-Q were examined using linear mixed models with random intercepts and unstructured covariance matrices. The change in each continuous outcome from the prior visit was used as the dependent outcome and the corresponding change in CDR staging from the prior visit was used as the independent outcome. The models were adjusted for patient characteristics (i.e., age, gender, race, education, primary type of residence) and clinical characteristics (i.e., cardiovascular disease, cerebrovascular disease, depression, and anxiety). Adjusted least square means and 95% confidence intervals (CIs) for each outcome were calculated. The probability of transition to long-term care facilities over time stratified by CDR staging of AD at the initial visit, was estimated using generalized estimating equation models, which controlled for patient and clinical characteristics. 

## Results

### Participant characteristics at the initial visit

A total of 28,220 participants were identified at an initial visit including 13,692 (48.5%) participants with unimpaired cognition, 7075 (25.1%) with MCI due to AD, and 4905 (17.4%), 1706 (6%), and 842 (3%) with mild, moderate, and severe AD dementia, respectively (Table 1). Of these 28,220 participants, 19,938 (70.7%) had at least one follow-up visit and 6270 (22.2%) had five follow-up visits. The mean age (SD) was 69.3 years (± 10.8) for the cognitively unimpaired and increased with more advanced AD stages to 76.3 years (± 11.1) for those with severe AD dementia. Participants tended to be female, White, with a high level of education, and living in single- or multi-family private residences. The proportion of participants who were dependent on others (required assistance with any degree of impairment in daily activities) were the lowest among cognitively unimpaired (1.9%), compared with 42, 84.8, 97.7, and 99.6% among those with MCI due to AD and mild, moderate, and severe AD dementia, respectively (Table 1). Approximately half (53%) of the study participants reported having a first-degree family member with cognitive impairment.

**Table 1:** Baseline demographics and clinical characteristics of participants

Variables, *n* (%)	Unimpaired cognition (*N* = 13,692)	MCI due to AD (*N* = 7075)	Mild AD dementia (*N* = 4905)	Moderate AD dementia (*N* = 1706)	Severe AD dementia (*N* = 842)
Age (years)					
Mean (SD)	69.3 (10.8)	73.0 (8.9)	74.0 (10.1)	75.7 (10.6)	76.3 (11.1)
Missing/unknown	0 (0)	0 (0)	0 (0)	0 (0)	0 (0)
BMI (kg/m^2^)					
Underweight, below 18.5	143 (1.0)	102 (1.4)	93 (1.9)	38 (2.2)	21 (2.5)
Healthy weight, 18.5–24.9	4433 (32.4)	2562 (36.2)	1740 (35.5)	572 (33.5)	236 (28.0)
Overweight, 25.0–29.9	4813 (35.2)	2482 (35.1)	1608 (32.8)	508 (29.8)	200 (23.8)
Obese, ≥ 30.0	3409 (24.9)	1338 (18.9)	810 (16.5)	290 (17.0)	75 (8.9)
Unknown	832 (6.1)	556 (7.9)	629 (12.8)	271 (15.9)	275 (32.7)
Not available or skipped	62 (0.5)	35 (0.5)	25 (0.5)	27 (1.6)	35 (4.2)
Sex					
Female	8976 (65.6)	3604 (50.9)	2698 (55.0)	1043 (61.1)	502 (59.6)
Race/ethnicity					
White	10,382 (75.8)	5574 (78.8)	3722 (75.9)	1134 (66.5)	608 (72.2)
Black/African American	1880 (13.7)	738 (10.4)	497 (10.1)	258 (15.1)	82 (9.7)
Hispanic	964 (7.0)	503 (7.1)	510 (10.4)	254 (14.9)	136 (16.2)
Asian	354 (2.6)	174 (2.5)	108 (2.2)	39 (2.3)	10 (1.2)
Others (with American Indian or Alaska Native and Native Hawaiian or other Pacific Islander)	97 (0.7)	73 (1.0)	56 (1.1)	18 (1.1)	5 (0.6)
Unknown	15 (0.1)	13 (0.2)	12 (0.2)	3 (0.2)	1 (0.1)
Level of education					
Less than high school	506 (3.7)	485 (6.9)	623 (12.7)	335 (19.6)	161 (19.1)
High school or GED	1737 (12.7)	1404 (19.8)	1210 (24.7)	441 (25.8)	206 (24.5)
Some college	2544 (18.6)	1202 (17.0)	826 (16.8)	283 (16.6)	120 (14.3)
Bachelor’s degree	3360 (24.5)	1646 (23.3)	1020 (20.8)	293 (17.2)	177 (21.0)
Master’s degree or doctorate	5461 (39.9)	2297 (32.5)	1183 (24.1)	335 (19.6)	157 (18.6)
Unknown	84 (0.6)	41 (0.6)	43 (0.9)	19 (1.1)	21 (2.5)
Level of independence					
Able to live independently	13,437 (98.1)	4093 (57.9)	745 (15.2)	40 (2.3)	3 (0.4)
Requires some assistance with complex activities	156 (1.1)	2612 (36.9)	2883 (58.8)	438 (25.7)	35 (4.2)
Requires some assistance with basic activities	62 (0.5)	278 (3.9)	1130 (23.0)	925 (54.2)	198 (23.5)
Completely dependent	12 (0.09)	18 (0.3)	83 (1.7)	291 (17.1)	601 (71.4)
Unknown	25 (0.2)	74 (1.0)	64 (1.3)	12 (0.7)	5 (0.6)
Primary type of residence					
Single- or multi-family private residence (apartment, condo, house)	12,835 (93.7)	6555 (92.7)	4417 (90.1)	1478 (86.6)	497 (59.0)
Retirement community or independent group living	594 (4.3)	295 (4.2)	201 (4.1)	49 (2.9)	7 (0.8)
Assisted living, adult family home, or boarding home	42 (0.3)	56 (0.8)	168 (3.4)	100 (5.9)	78 (9.3)
Skilled nursing facility, nursing home, hospital, or hospice	4 (0.03)	7 (0.10)	25 (0.5)	46 (2.7)	240 (28.5)
Others/unknown	217 (1.6)	162 (2.3)	94 (1.9)	33 (1.9)	20 (2.4)
First-degree family member with cognitive impairment					
Report of at least one	7496 (54.7)	3737 (52.8)	2472 (50.4)	843 (49.4)	445 (52.9)
None reported	4902 (35.8)	2560 (36.2)	1753 (35.7)	630 (36.9)	248 (29.5)
Unknown	1264 (9.2)	745 (10.5)	657 (13.4)	207 (12.1)	126 (15.0)
Not available or skipped	30 (0.2)	33 (0.5)	23 (0.5)	26 (1.5)	23 (2.7)
Comorbidities					
Cardiovascular disease	1991 (14.5)	1259 (17.8)	846 (17.2)	299 (17.5)	126 (15.0)
Stroke	63 (0.5)	54 (0.8)	64 (1.3)	26 (1.5)	29 (3.4)
Transient ischemic attack	118 (0.9)	106 (1.5)	89 (1.8)	36 (2.1)	14 (1.7)
Type 2 diabetes mellitus	512 (3.7)	279 (3.9)	137 (2.8)	41 (2.4)	11 (1.3)
Depression	1121 (8.2)	1375 (19.4)	1147 (23.4)	344 (20.2)	159 (18.9)
Anxiety	1542 (11.3)	2340 (33.1)	2148 (43.8)	822 (48.2)	333 (39.5)

### Association among changes in AD stages and outcomes of interest

The incremental changes in average FAS scores from the initial visit to visit 6 associated with each step of progression from cognitively unimpaired to MCI due to AD, MCI due to AD to mild AD dementia, mild to moderate AD dementia, and moderate to severe AD dementia were 6.2 (95% CI 3.8, 8.7), 16.9 (95% CI 14.4, 19.3), 13.8 (95% CI 11.3, 16.3), and 6.6 (95% CI 4.1, 9.1), respectively (Table 2). The corresponding average changes in FAS from the prior visit were 1.25 (95% CI 1.01, 1.49), 6.99 (95% CI 6.79, 7.19), 4.65 (95% CI 4.47, 4.83), and 1.46 (95% CI 1.27, 1.66), respectively (Table 3). The change in FAS from prior visits among participants who transitioned from moderate to severe AD dementia was similar to those who remained in the moderate stage.

**Table 2:** Changes in CDR from baseline to visit 6 and corresponding changes in selected outcomes of interest

Outcome	Change in scores from baseline to visit 6				
	Unimpaired cognition ∆ (95% CI)	MCI due to AD ∆ (95% CI)	Mild AD dementia ∆ (95% CI)	Moderate AD dementia ∆ (95% CI)	Severe AD dementia ∆ (95% CI)
NACC-FAS					
Unimpaired cognition	2.6 (0.2, 5.0)	6.2 (3.8, 8.7)	21.0 (18.5, 23.4)	30.4 (27.2, 33.6)	30.0 (28.4, 30.0)
MCI due to AD	–	5.2 (2.8, 7.7)	16.9 (14.4, 19.3)	22.5 (20.1, 25.0)	23.3 (20.8, 25.8)
Mild AD dementia	–	–	10.8 (8.2, 13.5)	13.8 (11.3, 16.3)	13.9 (11.4, 16.3)
Moderate AD dementia	–	–	–	6.3 (2.0, 10.7)	6.6 (4.1, 9.1)
Severe AD dementia	–	–	–	–	4.2 (1.5, 6.9)
GDS					
Unimpaired cognition	0.2 (− 1.3, 1.7)	0.9 (− 0.6, 2.4)	1.1 (− 0.5, 2.6)	2.2 (0.4, 4.0)	−
MCI due to AD	–	0.1 (− 1.4, 1.6)	− 0.2 (− 1.7, 1.3)	− 0.2 (− 1.7, 1.3)	0.6 (− 1.2, 2.3)
Mild AD dementia	–	–	− 0.4 (− 2.0, 1.1)	− 0.4 (− 1.9, 1.2)	− 0.3 (− 2.0, 1.3)
Moderate AD dementia	–	–	–	− 0.3 (− 2.5, 1.9)	0.9 (− 1.2, 2.9)
Severe AD dementia	–	–	–	–	6.9 (3.0, 10.8)
NPI-Q					
Unimpaired cognition	− 0.6 (− 2.4, 1.1)	0.8 (− 1.0, 2.6)	3.0 (1.2, 4.9)	4.5 (2.0, 6.9)	1.9 (− 1.7, 5.5)
MCI due to AD	–	0.1 (− 1.7, 1.9)	1.0 (− 0.8, 2.7)	3.3 (1.5, 5.1)	2.7 (0.9, 4.5)
Mild AD dementia	–	–	− 0.2 (− 2.0, 1.7)	2.1 (0.3, 3.9)	2.6 (0.8, 4.4)
Moderate AD dementia	–	–	–	1.2 (− 2.2, 4.5)	0.0 (− 1.9, 1.8)
Severe AD dementia	–	–	–	–	− 1.3 (− 3.5, 0.9)
MMSE					
Unimpaired cognition	1.1 (− 0.9, 3.1)	− 0.1 (− 2.2, 1.9)	− 5.2 (− 7.3, − 3.0)	− 10.7 (− 13.4, − 8.1)	–
MCI due to AD	–	− 0.6 (− 2.6, 1.5)	− 3.1 (− 5.1, − 1.0)	− 9.1 (− 11.2, − 7.0)	− 16.5 (− 18.7, − 14.3)
Mild AD dementia	–	–	− 2.3 (− 4.4, − 0.1)	− 6.2 (− 8.3, − 4.2)	− 14.5 (− 16.6, − 12.4)
Moderate AD dementia	–	–	–	− 2.1 (− 5.2, 0.9)	− 5.5 (− 7.9, − 3.1)
Severe AD dementia	–	–	–	–	− 8.9 (− 13.0, − 4.9)

**Table 3:** Changes in CDR stages from the prior visit and corresponding changes in selected outcomes of interest

Outcome	Change in scores from prior visit				
	Unimpaired cognition ∆ (95% CI)	MCI due to AD ∆ (95% CI)	Mild AD dementia ∆ (95% CI)	Moderate AD dementia ∆ (95% CI)	Severe AD dementia ∆ (95% CI)
NACC-FAS					
Unimpaired cognition	0.09 (− 0.05, 0.23)	1.25 (1.01, 1.49)	17.12 (15.92, 18.32)	20.52 (17.61, 23.44)	30.00 (27.62, 30.00)
MCI due to AD	–	1.41 (1.26, 1.56)	6.99 (6.79, 7.19)	11.85 (11.39, 12.32)	18.88 (17.90, 19.86)
Mild AD dementia	–	–	2.59 (2.43, 2.75)	4.65 (4.47, 4.83)	6.32 (5.99, 6.65)
Moderate AD dementia	–	–	–	1.44 (1.27, 1.61)	1.46 (1.27, 1.66)
Severe dementia	–	–	–	–	0.19 (0.03, 0.35)
GDS					
Unimpaired cognition	0.21 (0.14, 0.29)	0.60 (0.48, 0.72)	1.10 (0.49, 1.71)	2.02 (− 0.30, 4.33)	
MCI due to AD	–	0.14 (0.06, 0.21)	0.11 (0.01, 0.21)	0.05 (− 0.19, 0.30)	− 0.91 (− 2.22, 0.40)
Mild AD dementia	–	–	− 0.01 (− 0.09, 0.07)	0.01 (− 0.09, 0.12)	0.13 (− 0.17, 0.43)
Moderate AD dementia	–	–	–	0.05 (− 0.05, 0.15)	0.12 (− 0.08, 0.32)
Severe AD dementia	–	–	–	–	0.28 (0.02, 0.54)
NPI-Q					
Unimpaired cognition	0.43 (0.35, 0.51)	1.04 (0.84, 1.23)	3.93 (2.93, 4.94)	2.22 (− 0.59, 5.03)	1.34 (− 1.58, 4.26)
MCI due to AD	–	0.30 (0.21, 0.39)	1.06 (0.93, 1.19)	2.64 (2.29, 2.98)	3.99 (3.20, 4.79)
Mild AD dementia	–	–	0.12 (0.02, 0.21)	1.27 (1.14, 1.40)	3.06 (2.76, 3.36)
Moderate AD dementia	–	–	–	0.32 (0.20, 0.44)	1.01 (0.84, 1.18)
Severe AD dementia	–	–	–	–	− 0.51 (− 0.63, − 0.38)
MMSE					
Unimpaired cognition	0.01 (− 0.60, 0.61)	− 0.67 (− 1.30, − 0.04)	− 4.08 (− 5.22, − 2.95)	− 8.68 (− 12.03, − 5.32)	–
MCI due to AD	–	− 0.61 (− 1.22, − 0.01)	− 2.54 (− 3.15, − 1.92)	− 6.22 (− 6.94, − 5.51)	− 8.11 (− 9.64, − 6.58)
Mild AD dementia	–	–	− 1.43 (− 2.04, − 0.82)	− 4.25 (− 4.87, − 3.64)	− 8.62 (− 9.32, − 7.91)
Moderate AD dementia	–	–	–	− 2.18 (− 2.80, − 1.57)	− 4.95 (− 5.59, − 4.31)
Severe AD dementia	–	–	–	–	− 1.69 (− 2.32, − 1.06)
MoCA					
Unimpaired cognition	− 0.26 (− 0.85, 0.33)	− 1.44 (− 2.11, − 0.77)	− 0.51 (− 4.06, 3.04)	–	–
MCI due to AD	–	− 1.10 (− 1.70, − 0.51)	− 3.08 (− 3.70, − 2.45)	− 6.58 (− 7.54, − 5.63)	− 7.40 (− 12.64, − 2.15)
Mild AD dementia	–	–	− 2.16 (− 2.76, − 1.57)	− 4.32 (− 4.96, − 3.68)	− 6.05 (− 7.21, − 4.90)
Moderate AD dementia	–	–	–	− 2.34 (− 3.00, − 1.68)	− 3.21 (− 4.20, − 2.23)
Severe AD dementia	–	–	–	–	− 5.02 (− 6.56, − 3.48)

For NPI-Q, the significant average change from initial visit to visit 6 was observed in incremental transitions from mild to moderate AD dementia (2.1 [95% CI 0.3, 3.9]) (Table 2) while significant changes from prior visit were observed in incremental change from unimpaired cognition to MCI due to AD (1.04 [95% CI 0.84, 1.23]), MCI due to AD to mild AD dementia (1.06 [95% CI 0.93, 1.19]), mild to moderate AD dementia (1.27 [95% CI 1.14, 1.40]), and moderate to severe AD dementia (1.01 [95% CI 0.84, 1.18]) (Table 3). Significant changes in GDS from prior visits were also observed in participants who transitioned from unimpaired cognition to MCI due to AD (0.6 [95% CI 0.48, 0.72]) and from MCI due to AD to mild AD dementia (0.11 [95%, 0.01, 0.21]) (Table 3). The changes in GDS at visit 6 from the initial visit were not statistically significant.

Significant changes were also observed in MMSE and MoCA but in contrast, the larger changes were between the more severe stages. For the MMSE, the average changes from initial visit to visit 6 among participants who transitioned from unimpaired cognition to MCI due to AD, from MCI due to AD to mild AD dementia, from mild to moderate AD dementia, and from moderate to severe AD dementia were − 0.1 (95% CI − 2.2, 1.9), − 3.1 (95% CI − 5.1, − 1.0), − 6.2 (95% CI − 8.3, − 4.2), and − 5.5 (95% CI − 7.9, − 3.1), respectively (Table 2). The corresponding changes from prior visit in MMSE were − 0.67 (95% CI − 1.30, − 0.04) from unimpaired cognition to MCI due to AD, − 2.54 (95% CI − 3.15, − 1.92) from MCI due to AD to mild AD dementia, − 4.25 (95% CI − 4.87, − 3.64) from mild to moderate AD dementia, and − 4.95 (95% CI − 5.59, − 4.31) from moderate to severe AD dementia (Table 3). For MoCA, the corresponding observed average changes from the prior visit were − 1.44 (95% CI − 2.11, − 0.77), − 3.08 (95% CI − 3.70, − 2.45), − 4.32 (95% CI − 4.96, − 3.68), and − 3.21 (95% CI − 4.20, − 2.23), respectively (Table 3).

### Transition to long-term care facilities by CDR staging of AD at the initial visit

At the initial visit, the proportions of participants who were in long-term care facilities were 0.3, 0.9, 3.9, 8.6, and 37.8% among cognitively unimpaired, MCI due to AD, and mild, moderate, and severe AD dementia, respectively (Table 1). The adjusted odds of being admitted to long-term care facilities increased over time when participants were stratified by baseline CDR staging of AD; these are shown in Table 4. Overall, participants with MCI due to AD, mild, and moderate AD dementia had significantly higher odds of transition to long-term care facilities at visit 6 compared with the initial visit. Among participants with MCI due to AD, the adjusted odds of transition to long-term care facilities increased from 1.99 (95% CI 1.53, 2.58) at visit 2 to 8.78 (95% CI 6.31, 12.21) at visit 6, compared with the initial visit. The adjusted odds of transition to long-term care facilities increased from 2.25 (95% CI 1.94, 2.60) to 6.59 (95% CI 5.07, 8.56) compared with the initial visit among participants with mild AD dementia. The adjusted OR of transition to long-term care facilities increased from 2.11 (95% CI 1.73, 2.57) to 7.01 (95% CI 4.14, 11.86) compared with the initial visit among participants with moderate AD dementia.

**Table 4:** Adjusted analyses of being transitioned to long-term care facilities over time by CDR stage at initial visit

Odds of being transitioned to long-term care facilities	Unimpaired cognition		MCI due to AD		Mild AD dementia		Moderate AD dementia		Severe AD dementia	
	OR (95% CI)	*P*-value	OR (95% CI)	*P*-value	OR (95% CI)	*P*-value	OR (95% CI)	*P*-value	OR (95% CI)	*P*-value
Visit number										
Visit 2	1.20 (0.88, 1.63)	0.250	1.99 (1.53, 2.58)	< .0001	2.25 (1.94, 2.60)	< .0001	2.11 (1.73, 2.57)	< .0001	1.32 (1.06, 1.65)	0.014
Visit 3	1.35 (0.96, 1.91)	0.084	2.95 (2.20, 3.95)	< .0001	3.30 (2.78, 3.92)	< .0001	2.90 (2.22, 3.79)	< .0001	1.78 (1.28, 2.48)	0.001
Visit 4	1.31 (0.91, 1.88)	0.143	4.21 (3.10, 5.71)	< .0001	4.85 (4.00, 5.89)	< .0001	4.73 (3.39, 6.59)	< .0001	1.68 (1.06, 2.65)	0.026
Visit 5	1.25 (0.85, 1.84)	0.251	5.73 (4.15, 7.91)	< .0001	6.12 (4.90, 7.65)	< .0001	5.02 (3.26, 7.72)	< .0001	1.85 (0.98, 3.50)	0.059
Visit 6	1.49 (1.00, 2.22)	0.050	8.78 (6.31, 12.21)	< .0001	6.59 (5.07, 8.56)	< .0001	7.01 (4.14, 11.86)	< .0001	1.26 (0.53, 2.96)	0.602
Visit 1	Ref	Ref	Ref	Ref	Ref	Ref	Ref	Ref	Ref	Ref
Age	1.19 (1.16, 1.23)	< .0001	1.09 (1.07, 1.10)	< .0001	1.04 (1.03, 1.05)	< .0001	1.03 (1.01, 1.04)	0.001	1.01 (1.00, 1.03)	0.126
Race										
Black/African American	0.43 (0.20, 0.92)	0.029	0.45 (0.27, 0.74)	0.002	0.47 (0.31, 0.71)	0.000	0.42 (0.26, 0.68)	0.001	0.26 (0.12, 0.53)	0.000
Hispanic	0.99 (0.36, 2.68)	0.978	0.45 (0.19, 1.07)	0.070	0.42 (0.26, 0.69)	0.001	0.16 (0.08, 0.33)	< .0001	0.25 (0.13, 0.45)	< .0001
Others^a^	1.85 (0.49, 6.91)	0.363	0.50 (0.20, 1.26)	0.141	1.17 (0.63, 2.15)	0.622	0.30 (0.08, 1.19)	0.088	0.73 (0.19, 2.80)	0.645
White	Ref	Ref	Ref	Ref	Ref	Ref	Ref	Ref	Ref	Ref
Sex										
Male	0.74 (0.50, 1.09)	0.125	0.48 (0.38, 0.62)	< .0001	0.56 (0.46, 0.69)	< .0001	0.94 (0.68, 1.30)	0.708	0.95 (0.68, 1.34)	0.788
Female	Ref	Ref	Ref	Ref	Ref	Ref	Ref	Ref	Ref	Ref
Education										
Bachelor’s degree	1.43 (0.92, 2.22)	0.110	1.09 (0.80, 1.48)	0.576	1.01 (0.78, 1.31)	0.918	1.33 (0.85, 2.07)	0.215	1.31 (0.78, 2.19)	0.306
High school or GRE	1.49 (0.90, 2.47)	0.124	1.20 (0.87, 1.67)	0.267	0.75 (0.56, 0.99)	0.039	1.51 (0.98, 2.32)	0.060	1.00 (0.59, 1.68)	0.986
Less than high school	0.84 (0.29, 2.44)	0.747	0.93 (0.52, 1.69)	0.819	0.48 (0.30, 0.76)	0.002	1.52 (0.85, 2.72)	0.159	1.05 (0.54, 2.04)	0.875
Some college	1.01 (0.60, 1.71)	0.957	0.72 (0.50, 1.04)	0.079	0.93 (0.69, 1.24)	0.606	1.58 (0.97, 2.58)	0.066	0.82 (0.46, 1.45)	0.489
Master’s degree or doctorate	Ref	Ref	Ref	Ref	Ref	Ref	Ref	Ref	Ref	Ref
Cerebrovascular disease										
No	0.47 (0.28, 0.78)	0.004	0.70 (0.42, 1.18)	0.182	0.83 (0.54, 1.25)	0.365	0.57 (0.34, 0.95)	0.031	0.98 (0.54, 1.78)	0.945
Unknown/missing	1.10 (0.59, 2.05)	0.773	2.43 (1.38, 4.27)	0.002	2.63 (1.65, 4.19)	< .0001	1.46 (0.79, 2.68)	0.225	1.69 (0.68, 4.19)	0.256
Yes	Ref	Ref	Ref	Ref	Ref	Ref	Ref	Ref	Ref	Ref
Diabetes										
No	1.28 (0.58, 2.84)	0.535	1.49 (0.92, 2.42)	0.104	1.37 (0.80, 2.35)	0.259	4.11 (1.58, 10.72)	0.004	0.32 (0.10, 1.07)	0.065
Unknown/missing	1.38 (0.56, 3.43)	0.485	-		1.41 (0.77, 2.59)	0.262	3.43 (1.26, 9.38)	0.016	0.41 (0.12, 1.42)	0.159
Yes	Ref	Ref	Ref	Ref	Ref	Ref	Ref	Ref	Ref	Ref
Depression										
No	0.65 (0.44, 0.98)	0.039	1.00 (1.00, 1.00)		0.94 (0.78, 1.14)	0.549	0.88 (0.65, 1.20)	0.431	1.20 (0.81, 1.77)	0.373
Yes	Ref	Ref	Ref	Ref	Ref	Ref	Ref	Ref	Ref	Ref
Anxiety										
No	0.46 (0.31, 0.69)	0.000	1.03 (0.83, 1.26)	0.813	1.54 (1.33, 1.80)	< .0001	1.67 (1.31, 2.13)	< .0001	2.41 (1.81, 3.20)	< .0001
Unknown/missing	0.76 (0.36, 1.58)	0.462	2.72 (1.56, 4.75)	0.000	4.28 (2.77, 6.62)	< .0001	3.06 (1.66, 5.65)	0.000	4.70 (2.93, 7.54)	< .0001
Yes	Ref	Ref	Ref	Ref	Ref	Ref	Ref	Ref	Ref	Ref

^a^American Indian or Alaska Native and Native Hawaiian or other Pacific Islanders

*Ref* the reference standard to which other data are compared

### Total length of time spent at AD stages and annual transition probabilities in AD

The estimated average time spent in each AD stage was 3.2 years for MCI due to AD, and 2.2, 2.0, and 2.8 years for mild, moderate, and severe AD dementia, respectively. The annual probabilities of progressing from MCI due to AD to mild, moderate, and severe AD dementia, and death were 20.1, 4.3, 0.7, and 0.7%, respectively (Table 5). The corresponding annual probabilities of progressing from mild AD dementia to moderate AD dementia, severe AD dementia, and death were 26.6, 6.7, and 0.8%, respectively. The annual probability of dying among those with unimpaired cognition, MCI due to AD, mild, moderate, and severe AD dementia were 0.5, 0.7, 0.8, 5.3, and 25.3%, respectively and the cumulative annual transition probabilities to more advanced stages (including death) were 4.2, 25.8, 34.1, 36.4, and 25.3%, respectively (Table 5).

**Table 5:** Annual transition probabilities among unimpaired cognition and AD stages

AD stage	Annual transition probability (%)						
	Unimpaired cognition	MCI due to AD	Mild AD dementia	Moderate AD dementia	Severe AD dementia	Death	Cumulative probabilities of transition to any later stages
Unimpaired cognition	95.8%	3.2%	0.4%	0.1%	0.0%	0.5%	4.2%
MCI due to AD	4.8%	69.5%	20.1%	4.3%	0.7%	0.7%	25.8%
Mild AD dementia	0.1%	4.5%	61.3%	26.6%	6.7%	0.8%	34.1%
Moderate AD dementia	0.0%	0.1%	3.9%	59.6%	31.1%	5.3%	36.4%
Severe AD dementia	0.0%	0.0%	0.1%	2.0%	72.6%	25.3%	25.3%
Death	0.0%	0.0%	0.0%	0.0%	0.0%	100.0%	-

## Discussion

This study explored the transition in CDR staging of AD and change in relevant outcomes over time using data from a large sample of participants with unimpaired cognition and different stages of AD recruited at the NACC ADRCs across the USA. As anticipated, we found that changes in ADLs, neuropsychiatric features, and cognition were greatest among participants who transitioned from early AD stages, i.e., MCI due to AD or mild AD dementia to more advanced AD stages. Additionally, participants with more advanced AD stages were more likely to require assistance from others in daily living as observed in the baseline characteristics indicating a large difference in the proportion of patients being completely dependent and those living in nursing facilities among patients with mild vs. moderate/severe AD dementia. When compared with the initial visit, the odds of transitioning to long-term care facilities increased at each follow-up visit among participants with MCI due to AD, mild, and moderate AD dementia. Overall, this analysis provides estimates of relevant changes for several relevant domains across the AD spectrum, especially the earlier stages of MCI due to AD and mild AD dementia, which are the primary focus of most current therapeutic trials.

At the initial visit, almost half of the participants had unimpaired cognition while 25% had MCI due to AD and 25% had mild to severe AD dementia. Our analyses of change in AD stages from baseline to visit 6 and from prior visits revealed strong relationships with changes in ADLs measured by NACC-FAS. The change in FAS score was greatest among participants who transitioned from MCI due to AD or mild AD dementia to more advanced AD stages. These findings are consistent with a previous study using the NACC database reporting the minimally clinically important difference (MCID) of the NACC-FAS scores to be a 3–5-point increase among annual visits [10]. This indicated a meaningful decline in patient’s ADLs with increased AD severity [10]. According to our study, all transitions between stages resulted in a slightly wider range of average increase than the published change in the FAS, as the average change ranged from 1.46 (from moderate to severe AD dementia) to 6.99 (from MCI due to AD to mild AD dementia).

There was a strong association between the change in CDR stages and change in NPI-Q scores from the prior visit. The highest observed change in NPI-Q was among participants who transitioned from mild to moderate AD dementia. According to Mao et al., an MCID for the NPI-Q of 2.8 to 3.2 severity points within one month indicated a meaningful change [30]. Our study had a slightly lower change of NPI-Q score from baseline and from prior visits [30]. Compared with the participants in this study of long-term care facility residents, the individuals included in our study were predominantly in the early stages of AD, such as MCI and mild AD dementia. These findings suggest that early intervention and preventing or delaying the transition to more severe AD dementia may be crucial in reducing the negative impacts of AD.

The changes in GDS from prior visits among participants who transitioned from unimpaired cognition to MCI due to AD and from MCI due to AD to mild AD dementia were substantial. However, the largest increase in GDS was observed in those who remained in the severe AD dementia category. This may have occurred because the longer patients had severe symptoms, the more likely they have had depressive symptoms [31]. Although the GDS is an accurate screening test for depression for older adults in general, it might not be the most appropriate proxy measure of mood changes in adults with cognitive impairment [32]. Research on the MCID for GDS specifically in patients with AD dementia is limited. A previous study reported that the MCID for GDS varies across studies according to the patient population, methods used to determine MCID, and the version of GDS used as the GDS has 5-item, 15-item, and 30-item versions [33]. For example, the MCID was 1.6 to 1.9 in a study of adult patients with major depressive disorder in France using the 15-item version [34], while it was > 5 in patients in Japan using the 30-item version [35].

Our study found a severity-response relationship among stage-to-stage transition in AD and the change in cognition-related MMSE and MoCA scores among those who started in MCI due to AD or mild AD dementia stage and transitioned to more advanced stages from prior visit. As expected, the most severe transitions (e.g., from MCI or mild to severe AD dementia) were generally associated with the largest change in the scores of clinical scales. Our study also found that the change in clinical scores was larger in those who transitioned from early stages of AD (i.e., MCI due to AD or mild AD dementia) to moderate or severe AD dementia compared with those who transitioned from later stage (i.e., from moderate to severe AD dementia).

This study provides an update on annual probabilities of stage-to-stage transition in AD. We found that the cumulative annual transition probabilities to more advanced stages (including death) were 4.2, 25.8, 34.1, 36.4, and 25.3% for the cognitively unimpaired, MCI due to AD, and mild, moderate, and severe AD dementia, respectively. The annual probability of death was 25% for patients with severe AD dementia. Our results were similar to the annual progression rates reported by Davis et al. for MCI due to AD (22%) and moderate dementia (25%) stages, but differed for cognitively unimpaired (8%), mild dementia (25%), and annual probability of death in severe dementia (16%) at age 65 years [24]. These differences could be attributed to the application of two different methodologies to generate the transition probabilities. In our study, we did not account for baseline characteristics in the Markov model, while Davis et al. adjusted for baseline characteristics and used regression models to generate the probabilities. Another study using the NACC database estimated the progression rates across the spectrum of AD and reported that the annual transition probability to more severe dementia stages in symptomatic patients was 21.8, 36, and 28.6% from MCI due to AD to mild AD dementia, mild to moderate AD dementia, and moderate to severe AD dementia, respectively [19]. These figures are similar to those reported in the current study. Additionally, Vermunt et al. investigated the transition probabilities between different stages of AD and found that the probability of transitioning from MCI due to AD to mild AD dementia, from mild to moderate to severe AD dementia, and from moderate to severe AD dementia to death was 19.9, 20.0, and 16.4%, respectively [23]. The study by Vermunt et al. has the advantage of having a biomarker-confirmed population, while only a minority of the participants with data in the NACC dataset had a biomarker to confirm the diagnosis (~ 2%).

## Strengths and limitations

The strengths of this study include a large cohort of participants with unimpaired cognition, MCI due to AD and different stages of AD dementia from various ADRCs across the USA, and repeated measurements of AD and patient-relevant outcomes on an annual basis. The longitudinal design provides reliable estimates of stage-to-stage transition in AD. We considered the impact of CDR staging of AD on multiple outcomes including ADLs, neuropsychiatric features, cognition, and transitioning to long-term care facilities, thus providing a comprehensive picture of disease progression. However, the participating ADRCs are mostly academic institutions, which recruited patients with higher education and less advanced AD stages. People of racial/ethnic minorities are underrepresented in the study population. Thus, the study results may not be generalizable to the broader AD population.

Although the NACC database offers information on a large patient sample with long follow-up durations, there is a lack of use of biomarkers to confirm the diagnosis of AD, which may have affected the accuracy of our findings. Furthermore, the duration in a CDR stage at the initial visit was not available in the database and was not adjusted for in the analyses. As our analyses were on the population-level predictions rather than patient-level predictions, the Markov model was used. The Markov property assumes that all relevant information about future progression is contained in the current status. Therefore, the Markov model is computationally efficient and reliable for large datasets such as the NACC database.

While the data on patient-relevant outcomes were collected via validated instruments, some of these instruments were initially developed to detect changes only in early AD dementia stages. Thus, the observed relevant outcomes may not capture true changes among patients with advanced AD or those with milder stages. Patients with cognitive impairment and behavioral issues were also less likely to cooperate with clinical exams, and thus these instruments used to measure outcomes of interest may underestimate the true incidence and prevalence of behavioral disturbances and functional impairment.

## Conclusions

This study found that the changes in ADLs, neuropsychiatric features, and cognitive scores were the greatest among participants who transitioned from early AD stages, i.e., MCI due to AD or mild AD dementia to more advanced stages. We observed the highest odds of transitioning to long-term care facilities over time among participants with early stages, i.e., MCI due to AD or mild AD dementia. These findings suggest that early intervention in people with MCI due to AD or mild AD dementia may provide significant value in delaying the progression to later stages. These findings also assist in translating clinical trial outcomes using the CDR to a range of other commonly used instruments. The transition probabilities reflect AD progression which can be used to inform future economic models. Future research is warranted to understand the characteristics and features of AD disease progression and the impact on individuals experiencing AD dementia.

## Data Availability

The datasets used and/or analyzed during the current study are available from the corresponding author upon reasonable request.

## Abbreviations

AD: Alzheimer’s disease
ADL: Activity of daily living
ADRC: Alzheimer’s Disease Research Center
CDR: Clinical Dementia Rating
FAS: Functional Assessment Scale
GDS: Geriatric Depression Scale
MCI: Mild cognitive impairment
MCID: Minimally clinically important difference
MMSE: Mini-Mental State Exam
MoCA: Montreal Cognitive Assessment
NACC: National Alzheimer’s Coordinating Center
NIA: National Institute on Aging
NPI-Q: Neuropsychiatric Inventory Questionnaire
SD: Standard deviation
UDS: Uniform Data Set
